# Use of historical high-sensitivity cardiac troponin T levels to rule out myocardial infarction

**DOI:** 10.1136/openhrt-2021-001682

**Published:** 2021-05-14

**Authors:** Andreas Roos, Martin J Holzmann

**Affiliations:** 1 Department of Emergency and Reparative Medicine, Karolinska University Hospital, Huddinge, Karolinska Universitetssjukhuset, Stockholm, Sweden; 2 Department of Medicine Solna, Karolinska Institute, Stockholm, Sweden

**Keywords:** biomarkers, myocardial infarction, chest pain

## Abstract

**Objective:**

Several high-sensitivity cardiac troponin (hs-cTn)-based strategies exist for rule-out of myocardial infarction (MI). It is unknown whether historical hs-cTnT concentrations can be used. This study aim to evaluate the performance of a rule-out strategy based on the European Society of Cardiology (ESC) 0/1-hour algorithm, using historical hs-cTnT concentrations.

**Methods:**

All visits among patients with chest pain in the emergency department at nine different hospitals in Sweden from 2012 to 2016 were eligible (221 490 visits). We enrolled patients with a 0-hour hs-cTnT of <12 ng/L, a second hs-cTnT measured within 3.5 hours, and ≥1 historical hs-cTnT available. We calculated the risks of MI and all-cause mortality using two rule-out strategies: (1) a delta hs-cTnT of <3 ng/L between the 0-hour hs-cTnT and the second hs-cTnT (modified ESC algorithm) and (2) a historical hs-cTnT <12 ng/L and a delta hs-cTnT of <3 ng/L in relation to the 0-hour hs-cTnT (historical-hs-cTnT algorithm).

**Results:**

A total of 8432 patients were included, of whom 84 (1.0%) had an MI. The modified ESC algorithm triaged 8100 (96%) patients toward ruled-out, for whom 30-day MI risk and negative predictive value (NPV) for MI (95% CI) were 0.4% (0.3% to 0.6%) and 99.6% (99.4% to 99.7%), respectively. The historical-hs-cTnT algorithm ruled out 6700 (80%) patients, with a 30-day MI risk of 0.5% (0.4% to 0.8%) and NPV of 99.5% (99.2% to 99.6%).

**Conclusions:**

The application of algorithm resulted in similar MI risk and NPV to an established algorithm. The usefulness of historical hs-cTnT concentrations should merit further attention.

**Key questions**

**What is already known about this subject?**

- No data exist regarding the appropriate use of high-sensitivity cardiac troponin (hs-cTn) levels recorded at prior visits in patients with chest pain in the emergency department (ED). We investigated if hs-cTnT levels from historical visits could be integrated in an hs-cTn-based algorithm for rule-out of myocardial infarction (MI) in the ED.

**What does this study add?**

- The low risk of MI and death associated with the combination of a low historical hs-cTnT and a low 0-hour hs-cTnT in the ED indicate that information about historical hs-cTnT values may be clinically useful.

**How might this impact on clinical practice?**

- Clinicians currently have no guidance about how to properly use data on historical hs-cTnT concentrations in clinical care. Our findings indicate that such information may be of prognostic value, and its potential use should be further explored.

## Introduction

The use of high-sensitivity cardiac troponin (hs-cTn) assays has improved the early diagnosis of myocardial infarction (MI) and allowed the development of several novel cTn-based strategies that permit a safe and rapid rule-out of MI in the emergency department (ED).^1–3^ The European Society of Cardiology (ESC) 0/1-hour algorithm uses the hs-cTnT concentration at presentation and the absolute change within 1 hour to rapidly triage patients to either rule-out or rule-in of MI.^4^ Accordingly, a detectable 0-hour hs-cTnT concentration of <12 ng/L, and a concurrent absolute 1-hour change of <3 ng/L, can be used to safely rule out MI.^4–8^

Historical hs-cTn levels (hs-cTn concentrations measured during prior hospital visits) are commonly available for consideration in patients who present at the ED with symptoms that are suggestive of an evolving MI. However, no data exist regarding the prognostic value and appropriate use of historical hs-cTn levels such as in the context of a biomarker-based rule-out algorithm.

We therefore conducted a large observational cohort study to investigate the diagnostic performance of an established biomarker-based algorithm for rule-out of MI in patients with chest pain in the ED with any historical hs-cTnT value available and a 0-hour hs-cTnT of <12 ng/L, and assessed the performance of a historical-hs-cTnT algorithm that integrated the use of a historical hs-cTnT concentration as the 0-hour hs-cTnT level.

## Methods

### Study population

**Figure 1:**
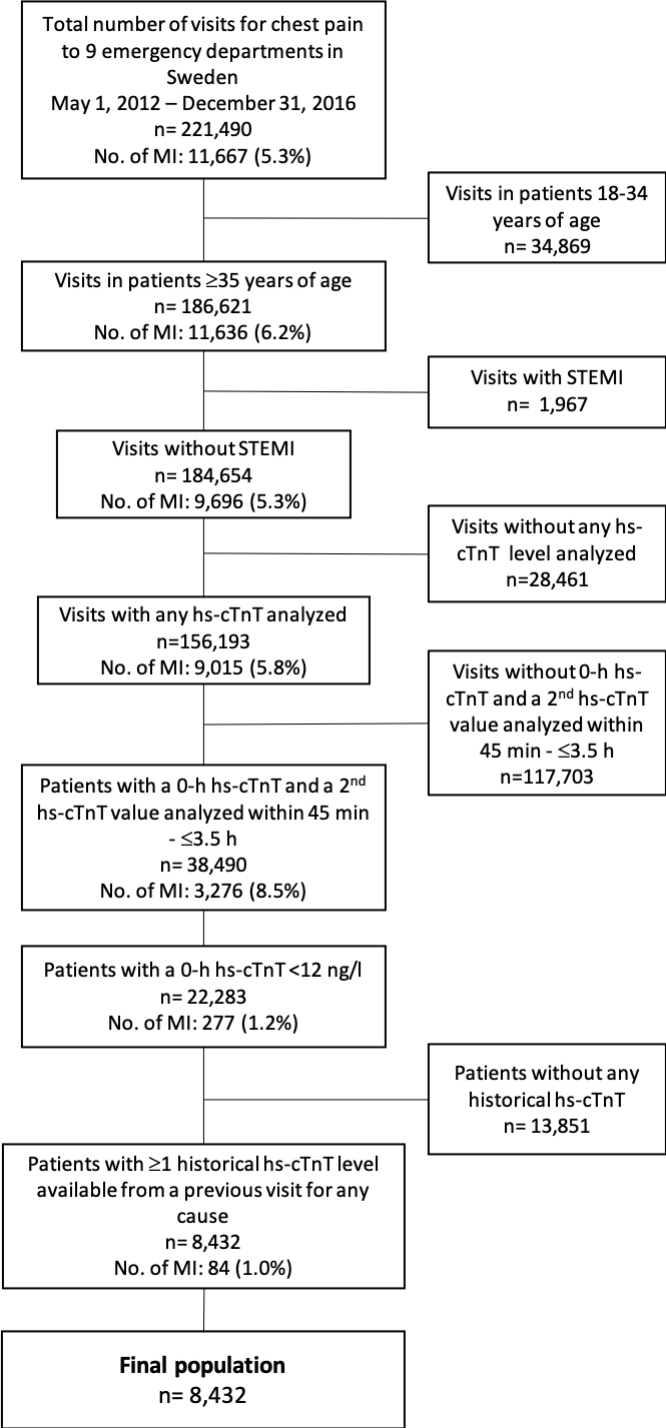
Selection of the study population. hs-cTnT, high-sensitivity cardiac troponin T; STEMI, ST-segment elevation myocardial infarction.

All patients >35 years of age with chest pain as their principal complaint in the ED at nine different hospitals in Sweden from 1 May 2012 to 31 December 2016, were eligible for inclusion (186 621 visits) (figure 1 and online supplemental table 1). Patients with ST-segment elevation MI associated with the visit were excluded (n=1967). From the remaining patients, we included all of those who had a first hs-cTnT concentration during the visit (0-hour hs-cTnT) of <12 ng/L and a second hs-cTnT level measured between 45 min and 3.5 hours subsequently, and for whom there was also ≥1 available hs-cTnT level from a previous visit for any cause (n=8432).

10.1136/openhrt-2021-001682.supp1 Supplementary data

The study was conducted according to Strengthening the Reporting of Observational Studies in Epidemiology guidelines.

### Data sources

Eligible patients were identified from the hospitals’ local administrative databases, which contain information regarding all ED visits. All patients visiting the ED at all sites during the study period were triaged by an attending nurse according to the standardised triage module Rapid Emergency Triage and Treatment System. This system includes standardised classification of primary symptoms in the ED, including chest pain, which together with information on patient history and vital parameters at presentation provides guidance for appropriate and effective prioritisation.

Laboratory data were obtained from each hospital’s IT Department. The data were then sent to the National Board of Health and Welfare, to retrieve information regarding comorbidities, current medications and outcomes from the National Patient Register, the Cause of Death Register and the Prescribed Drug Register.^9 10^

Hs-cTnT concentrations were measured using the Elecsys 2010 system (Roche Diagnostics, Mannheim, Germany). The assay has limit of blank of 3 ng/L, a recommended limit of detection of 5 ng/L, a coefficient of variation of <10% at 13 ng/L, and a 99 th percentile cut-off value of 14 ng/L.^11^ In hospitals in Stockholm, hs-cTnT concentrations <5 ng/L were not reported as absolute values, but these samples were assigned a level of 5 ng/L to calculate delta values between hs-cTnT concentrations.

### Definitions

The index date was defined as the day of the ED visit at which there was a primary report of chest pain. The 0-hour hs-cTnT level was defined as the first hs-cTnT concentration measured on the index date (ie, the admission hs-cTnT), and the second hs-cTnT concentration was defined as an hs-cTnT concentration measured between 45 min and 3.5 hours after the 0-hour hs-cTnT concentration. The historical hs-cTnT concentration was defined as the most recent hs-cTnT level measured >7 days before the 0-hour hs-cTnT concentration. The delta hs-cTnT was defined as the absolute change in hs-cTnT concentration between the 0-hour hs-cTnT and the second hs-cTnT concentration. Comorbidities were defined as discharge diagnoses prior to the ED visit that were coded according to the tenth version of the International Classification of Disease (ICD-10) in the National Patient Register, with the exception of diabetes, which was defined as the ongoing use of any hypoglycaemic agent. Ongoing use of medication was defined as ≥2 filled prescriptions during the year preceding the index date.

### Rule-out algorithms

Two hs-cTnT-based algorithms using delta hs-cTnT concentrations to triage patients toward rule-out were evaluated: a ESC-based algorithm, and a historical-hs-cTnT algorithm with the incorporation of a historical hs-cTnT level. With the modified ESC algorithm, the rule-out criteria were defined as a delta hs-cTnT <3 ng/L, according to the ESC 0/1-hour algorithm for triage toward rule-out of MI.^4^ With the historical-hs-cTnT algorithm, the 0-hour hs-cTnT level was replaced by the historical hs-cTnT concentration, and the 0-hour hs-cTnT concentration was used as the second hs-cTnT value. The rule-out criteria for this algorithm were defined as a historical hs-cTnT concentration of <12 ng/L, and a delta hs-cTnT of <3 ng/L (the change in hs-cTnT between the historical hs-cTnT value and the former 0-hour hs-cTnT value).

### Outcomes

MI was defined as a discharge diagnosis in the National Patient Register according to the ICD-10 codes I21 or I22 in the primary position in immediate conjunction, or within 30 days (for patients discharged from the ED) of the index date. All-cause mortality was defined as death from any cause that was registered in the Cause of Death register within 30 days of the index date.

### Statistical analysis

For both algorithms, we calculated absolute risks, negative predictive values (NPVs), negative likelihood ratios (LR^−^s) and sensitivities with 95% CIs for MI and all-cause mortality within 30 days of the ED visit. The LR^−^s were calculated to assess how much the application of the algorithms alter the odds of MI. Thus, the values could be explained as the change in the odds of having an MI in patients being triaged toward rule-out.

All the analyses were conducted separately for men and women. In a subgroup analysis of the modified ESC algorithm, patients were also categorised according to the time between the 0-hour hs-cTnT and second hs-cTnT measurements, into either an early resampling (second hs-cTnT measured between 45 min and 2 hours) or late resampling (second hs-cTnT level measured between 2 hours and 3.5 hours) group.^12^ In an additional analysis of the performance of the historical-hs-cTnT algorithm, we categorised patients into those who had a historical hs-cTnT concentration that had been measured within the preceding year, and those for which a measurement had been made >1 year prior to the 0-hour hs-cTnT concentration. In a supplemental analysis of the historical-hs-cTnT algorithm, we included all patients both with and without a second hs-cTnT measurement at the index visit. We used SAS V.9.4 software (SAS Institute) and R V.4.0.1, R Foundation for Statistical Computing (URL http://www.R-project.org/).

### Patient and public involvement

Patient or public involvement was not feasible or appropriate for this study.

## Results

### Study population

**Figure 2:**
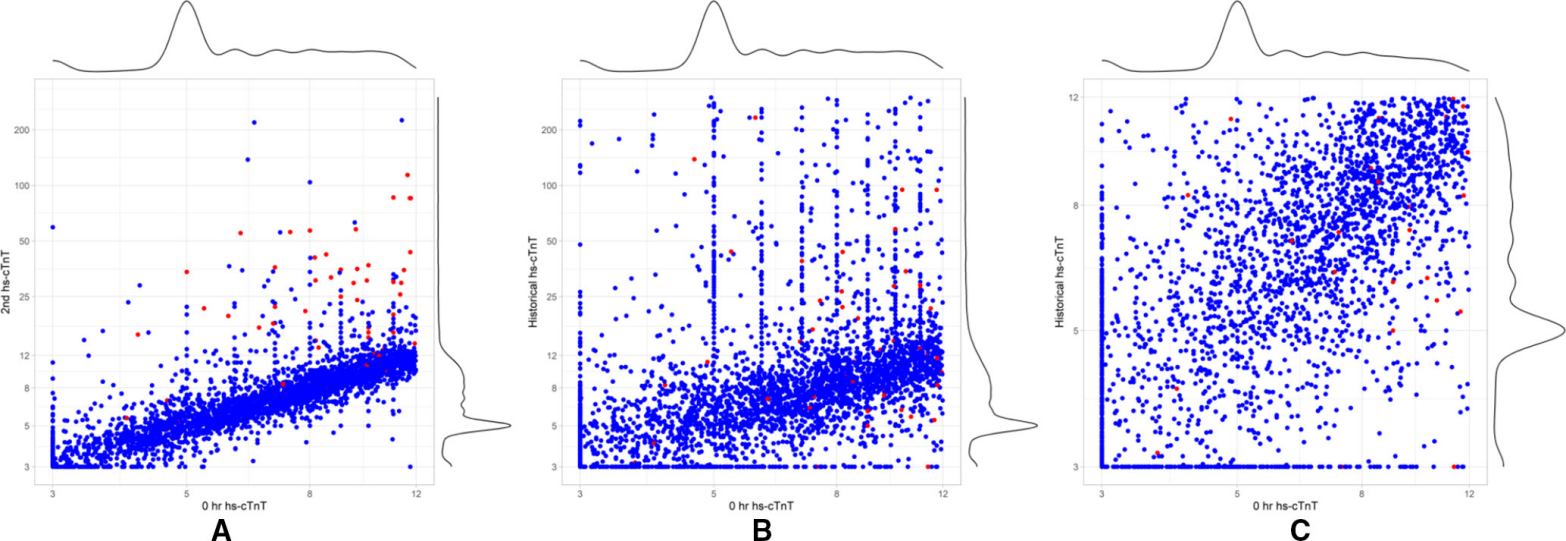
Histogram and scatter plots of historical hs-cTnT, 0-hour hs-cTnT and second hs-cTnT concentrations. Red dots indicate patients who experienced an MI and blue dots indicate patients who did not. (A) 0-hour hs-cTnT concentration vs second hs-cTnT concentration. (B) Historical hs-cTnT concentration vs 0-hour hs-cTnT concentration. (C) Historical hs-cTnT concentration of <12 ng/L vs 0-hour hs-cTnT concentration. hs-cTnT, high-sensitivity cardiac troponin T; MI, myocardial infarction.

In total, 8432 patients were included, of whom 84 (1.0%) had an MI within 30 days of the ED visit (table 1). Patients who had an MI were older, more likely to be men, and to have diabetes and have had a prior MI, and to have more medications than patients without MI (table 1). The mean delta hs-cTnT values were 13.8±20 ng/L and 0.3±4.3 ng/L for patients who did or did not have an MI, respectively (table 1). The historical hs-cTnT concentrations, and the 0-hour hs-cTnT and second hs-cTnT concentrations at the index visit are displayed in figure 2.

**Table 1:** Baseline characteristics

	All patients	Patients with MI	Patients without MI
No of patients	8432	84	8348
Age, years (SD)	61.7 (12.7)	66.5 (12.3)	61.7 (12.7)
Women, n (%)	4163 (49)	29 (35)	4134 (50)
**Index visit data**			
0-hour hs-cTnT concentration (ng/L), median (IQR)	6.0 (3.3)	8.8 (3.6)	6.0 (3.3)
Delta hs-cTnT (ng/L), mean (SD)*	0.4 (4.9)	13.8 (20.0)	0.3 (4.3)
Early retest (45 min to <2 hours), n (%)	3847 (46)	26 (31)	3821 (46)
Late retest (2 hours to ≤3.5 hours), n (%)	4585 (54)	58 (69)	4527 (54)
Time from 0-hour hs-cTnT to second hs-cTnT measurement, min, median (IQR)	133 (101)	175 (94)	132 (101)
Early retest group (45 min to <2 hours), n (%)	77 (27)	75 (31)	77 (27)
Late retest group (2 to ≤3.5 hours), n (%)	179 (28)	181 (18)	178 (28)
Historical hs-cTnT concentration (ng/L), median (IQR)	6.3 (4.6)	9.0 (11.8)	6.3 (4.5)
Time from historical hs-cTnT to 0-hour hs-cTnT measurement, days, median (IQR)	207 (464)	169 (531)	208 (463)
**Comorbidities**			
Prior stroke, n (%)	506 (6.0)	3 (3.6)	503 (6.0)
Prior MI, n (%)	2017 (24)	44 (52)	1973 (24)
COPD, n (%)	538 (6.4)	6 (7.1)	532 (6.4)
Prior heart failure, n (%)	345 (4.1)	3 (3.6)	342 (4.1)
Diabetes, n (%)	1012 (12)	23 (27)	989 (12)
Chronic kidney disease (eGFR <60 mL/min/1.73 m^2^), n (%)	65 (0.8)	2 (2.4)	63 (0.8)
Peripheral arterial disease, n (%)	90 (1.1)	6 (7.1)	84 (1.0)
Prior coronary angiography, n (%)	3100 (37)	53 (63)	3047 (37)
Prior revascularisation, n (%)	2375 (28)	44 (52)	2331 (28)
**Medication**			
Aspirin, n (%)	3046 (36)	51 (61)	2995 (36)
P2Y12 inhibitor†, n (%)	1243 (15)	27 (32)	1216 (15)
Beta-blockers, n (%)	3930 (47)	57 (68)	3873 (46)
ACE/ARB, n (%)	3635 (43)	50 (60)	3585 (43)
Statins, n (%)	3450 (41)	53 (63)	3397 (41)
OAC, n (%)	1030 (12)	9 (11)	1021 (12)
Warfarin, n (%)	687 (8.1)	8 (9.5)	679 (8.1)
NOAC, n (%)	383 (4.5)	2 (2.4)	381 (4.6)

*Delta hs-cTnT between the 0-hour hs-cTnT and second hs-cTnT measurements.

†P2Y12 inhibitor, including clopidogrel, ticagrelor or prasugrel.

ACEi/ARB, ACE inhibitor/angiotensin receptor blocker; COPD, chronic obstructive pulmonary disease; hs-cTn, high-sensitivity cardiac troponin; MI, myocardial infarction; NOAC, new oral anticoagulant; OAC, oral anticoagulants.

### Myocardial infarction

**Figure 3:**
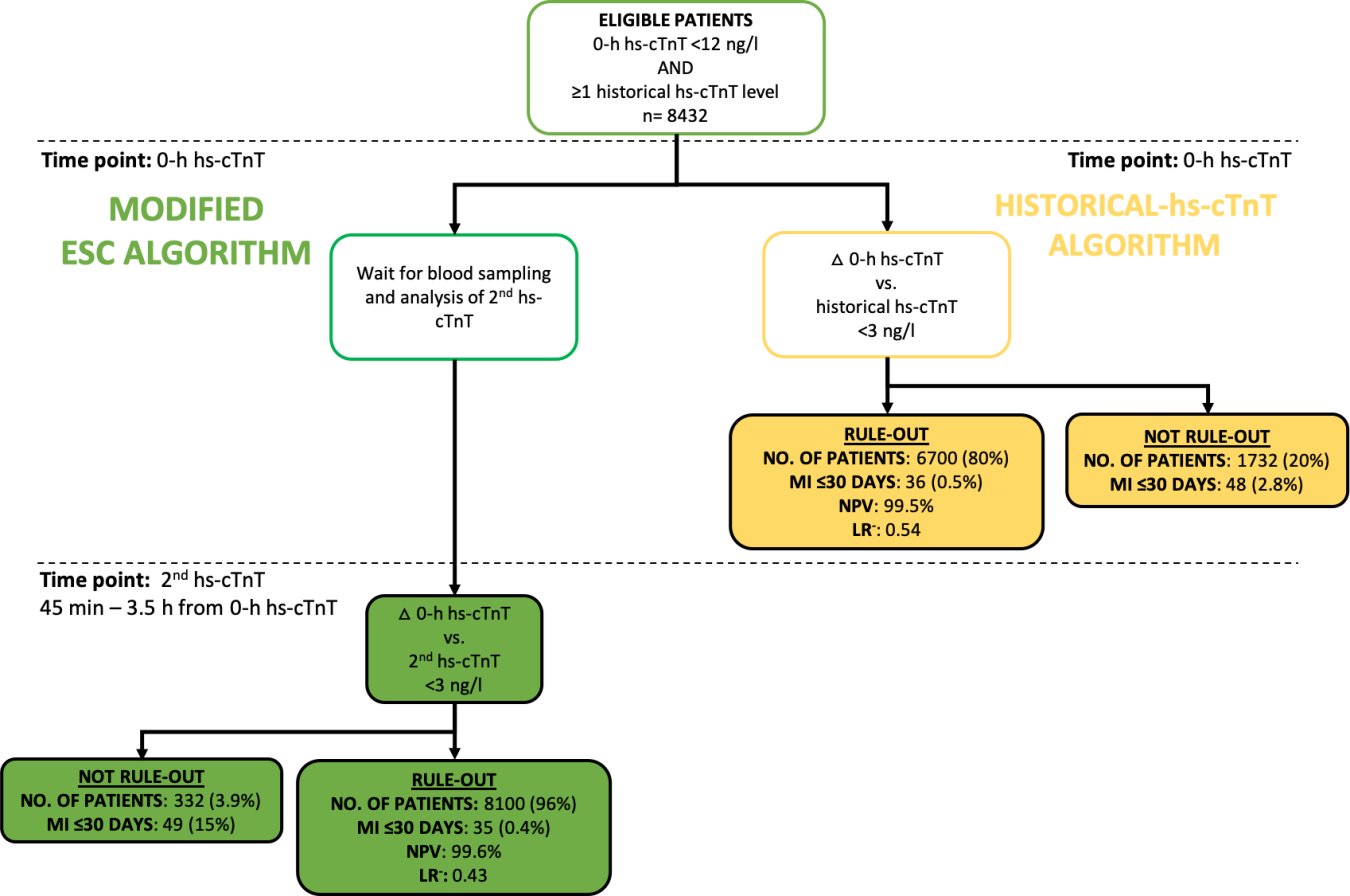
Comparison of two algorithms used to rule out myocardial infarction. All patients within the final population were eligible for both algorithms. The modified ESC algorithm triaged patients toward rule-out if the delta change between the 0-hour hs-cTnT concentration and the second hs-cTnT concentration measured 45 min to 3.5 hours from the 0-hour hs-cTnT was <3 ng/L. The historical-hs-cTnT algorithm triaged patients directly towards rule-out if the delta change between the historical hs-cTnT concentration and the 0-hour hs-cTnT concentration was <3 ng/L. ESC, European Society of Cardiology; hs-cTnT, high-sensitivity cardiac troponin T; LR^−^, negative likelihood ratio; MI, myocardial infarction; NPV, negative predictive value.

The modified ESC algorithm triaged 8100 (96%) of the patients toward rule-out (table 2 and figure 3). In total, 84 (1.0%) MIs occurred among all the eligible patients, which corresponded to 30-day MI risks of 0.4% and 14.8% for patients in whom MI was ruled out and not ruled out, respectively (table 2 and online supplemental table 2). The NPV, LR^−^ and sensitivity for MI of the algorithm were 99.6% (99.4%–99.7%), 0.43 (0.27–0.55) and 58.3% (47.1%–68.8%), respectively (table 2).

**Table 2:** Performance of a modified ESC algorithm and a historical-hs-cTnT algorithm with the use of a historical hs-cTnT value as the 0-hour value to rule out MI

No of eligible patients	8432 (100)	
MI ≤30 days after the index visit, n (%)	84 (1.0)	
**Algorithm using hs-cTnT measured at the same visit** (**modified ESC algorithm**)		
Total no of patients ruled-out, n (%)	TN (%)	FN (%)
8100 (96)	8065 (99.6)	35 (0.4)
Total no of patients not ruled-out, n (%)	TP (%)	FP (%)
332 (3.9)	49 (15)	283 (85)
**Rule-out**		
No of events, (%)	35 (0.4)	
30-day risk of MI (95% CI)	0.4% (0.3% to 0.6%)	
NPV, % (95% CI)	99.6 (99.4 to 99.7)	
LR^−^ (95% CI)	0.43 (0.27 to 0.55)	
Sensitivity, % (95% CI)	58.3 (47.1 to 68.8)	
**Algorithm using a historical hs-cTnT value as the 0-hour hs-cTnT value** (**historical-hs-cTnT algorithm**)		
Total no of patients ruled-out, n (%)	TN (%)	FN (%)
6700 (80)	6664 (99.5)	36 (0.5)
Total no of patients not ruled-out, n (%)	TP (%)	FP (%)
1732 (20)	48 (2.8)	1684 (97)
**Rule-out**		
No of events, (%)	36 (0.5)	
30-day risk of MI (95% CI)	0.5% (0.4% to 0.8%)	
NPV, % (95% CI)	99.5 (99.2 to 99.6)	
LR^−^ (95% CI)	0.54 (0.34 to 0.68)	
Sensitivity, % (95% CI)	57.1 (45.9 to 67.7)	

ESC, European Society of Cardiology; FN, false negative; FP, false positive; hs-cTN, high-sensitivity cardiac troponin; LR−, negative likelihood ratio; MI, myocardial infarction; NPV, negative predictive value; TN, true negative; TP, true positive.

In total, 6700 (80%) of all eligible patients were ruled out with the historical-hs-cTnT algorithm (figure 3 and table 2), corresponding to 3% (6700/221 490) of the total number of visits with chest pain in the ED, and 4.3% (6700/156 193) of patients with at least one hs-cTnT measured during the index visit (figure 1). The 30-day MI risk was 0.5% in patients for whom MI was ruled out, whereas it was 4.6% among all the other eligible patients (table 2 and online supplemental table 2). The NPV and sensitivity for MI using the historical-hs-cTnT algorithm were 99.5% (99.2%–99.6%) and 57.1% (45.9%–67.7%), respectively, and the LR^−^ was 0.54 (0.34–0.68).

The majority of MIs occurred in men (65%), and point estimates indicated higher NPV and lower LR^−^ in women compared with men, with both algorithms (online supplemental table 3). In total, 197 patients for whom MI was not ruled out using the modified ESC algorithm were ruled out with the historical-hs-cTnT algorithm, which represented 2.9% of the total number of patients ruled out using this algorithm (figure 3). In total, 17 MIs that occurred within 30 days of the index date were ‘missed’, meaning that MI was ruled out only using the historical-hs-cTnT algorithm (0.3% of all the patients triaged toward rule-out with this protocol).

### All-cause mortality

Thirteen (0.2%) deaths occurred within 30 days of the index date (table 3). The 30-day mortality of patients triaged toward rule-out using the modified ESC algorithm was 0.1%, and it was 0.3% in all the other patients, among whom only one death occurred (table 3 and online supplemental table 4). The NPV and LR^−^ for all-cause mortality using the modified ESC algorithm were 99.9% (99.7%–99.9%) and 0.96 (0.28–1.04), respectively (table 3). The corresponding figures for the historical-hs-cTnT algorithm were 99.9% (99.8%–100%) and 0.39 (0.04–1.01). No deaths were ‘missed’ with the historical-hs-cTnT algorithm, meaning that no deaths occurred among those ruled-out only by this algorithm (online supplemental table 4). The diagnostic performances of each algorithm were similar for men and women (online supplemental table 3).

**Table 3:** Performance of a modified ESC algorithm and a historical-hs-cTnT algorithm with the use of a historical hs-cTnT value as the 0-hour value for the prediction of all-cause mortality

No of eligible patients	8432 (100)	
Death ≤30 days after the index visit, n (%)	13 (0.2)	
**Algorithm using hs-cTnT measured at the same visit** (**modified ESC algorithm**)		
Total no of patients ruled-out, n (%)	TN (%)	FN (%)
8100 (96)	8088 (99.9)	12 (0.1)
Total no of patients not ruled-out, n (%)	TP (%)	FP (%)
332 (3.9)	1 (0.3)	331 (99.7)
**Rule-out**		
No of events, (%)	12 (0.1)	
30-day risk of all-cause mortality (95% CI)	0.1% (0.1% to 0.3%)	
NPV, % (95% CI)	99.9 (99.7 to 99.9)	
LR^−^ (95% CI)	0.96 (0.28 to 1.04)	
Sensitivity, % (95% CI)	7.7 (0.4 to 37.9)	
**Algorithm using a historical hs-cTnT value as the 0-hour hs-cTnT value** (**historical-hs-cTnT algorithm**)		
Total no of patients ruled-out, n (%)	TN (%)	FN (%)
6700 (79)	6696 (99.9)	4 (0.1)
Total no. of patients not ruled-out, n (%)	TP (%)	FP (%)
1732 (20)	9 (0.5)	1723 (99.5)
**Rule-out**		
No of events, (%)	4 (0.1)	
30-day risk of all-cause mortality (95% CI)	0.1% (0.0% to 0.2%)	
NPV, % (95% CI)	99.9 (99.8 to 100)	
LR^−^ (95% CI)	0.39 (0.04 to 1.01)	
Sensitivity, % (95% CI)	69.2 (38.9 to 89.6)	

ESC, European Society of Cardiology; FN, false negative; FP, false positive; hs-cTN, high-sensitivity cardiac troponin; LR−, negative likelihood ratio; NPV, negative predictive value; TN, true negative; TP, true positive.

### Subgroup analysis

MI was ruled out using the modified ESC algorithm in similar proportions of patients who underwent early or late resampling of their hs-cTnT concentration (online supplemental table 5). The majority of MIs occured in patients who underwent late resampling (69%), of whom 1.3% had an MI within 30 days, and the corresponding figure was 0.7% for the early resampling group. The diagnostic performance of the modified ESC algorithm, in terms of the NPVs for both MI and all-cause mortality, did not substantially differ between patients who underwent early or late resampling (online supplemental tables 5 and 6, respectively).

Two out of three patients (65%) had a historical hs-cTnT concentration that had been recorded within the year preceding the index date (online supplemental table 5). Among the patients for whom MI had been ruled out using this algorithm, the 30-day MI risk was slightly lower, and the corresponding NPV higher, than in those who had a historical hs-cTnT concentration that had been recorded >1 year before the index date. The diagnostic performance for all-cause mortality did not differ between the subgroups (online supplemental table 6).

The perfomance of the historical-hs-cTnT algorithm for MI and all-cause mortality when patients with only one hs-cTnT concentration measured during the index visit also were included (ie, the 0-hour hs-cTnT level), was similar to that observed in the main analyses (online supplemental table 7).

## Discussion

In a large cohort of patients with chest pain in the ED and a hs-cTnT level at presentation of <12 ng/L who all had historical hs-cTnT values available, we found that MI within the subsequent 30 days could be safely ruled out using an established biomarker-based algorithm. The application of a historical-hs-cTnT algorithm, in which the 0-hour hs-cTnT concentration was replaced by a historical hs-cTnT value, was also associated with a low risk of MI in patients triaged toward rule-out. Our findings indicate that the incorporation of historical hs-cTnT levels in clinical algorithms could influence decisions in a large number of patients with chest pain, in whom the need for serial hs-cTnT testing may be reduced and consequently the length of stay in the ED. Overall, four in five patients with hs-cTnT concentrations<12 ng/L at presentation and who had prior hs-cTnT concentrations available were able to be ruled out without the need for a second test, with similar diagnostic performance as the modified ESC algorithm. A significant reduction of the length of stay in the ED could ultimately lead to a lowering of the levels of ED crowding, which is a major problem worldwide with negative consequences on patients, staff and the healthcare system.

Historical hs-cTnT levels are commonly available for patients who present with chest pain in the ED. In the present cohort, 40% of all the patients with a 0-hour hs-cTnT of <12 ng/L had had an hs-cTnT concentration recorded at a previous visit. However, if such information could be used to enhance rule-out decisions should be considered with caution. First, cTn concentrations should always be evaluated in conjunction with all the other medical information available, including clinical parameters and electrocardiographic findings. Patients with hs-cTnT levels in the lower range without a temporal change, but who have been evaluated as being at high risk of MI or other acute cardiovascular event, should undergo careful clinical assessment, including further diagnostic testing and clinical monitoring if necessary.^4 12^ Second, it is important to emphasise that only patients with a 0-hour hs-cTnT of <12 ng/L were included in the present study; therefore, there was a low incidence of MI, which was attributable to the high NPVs (particularly as sensitivities also were low with wide CIs). The moderate LRs− for both pathways indicate only a slight difference between pre-test and post-test probabilities (approaching pre-test and post-test odds, respectively), which also may be related to the low MI incidence. Algorithm development with optimised rule-out decision thresholds using historical hs-cTnT levels would need to be derived using cohorts in which a large number of MIs had occurred, without restricting eligibility by 0-hour hs-cTnT levels, and should be thoroughly validated.^13^

We found a similarly low 30-day MI risk and high NPV for MI in patients for whom MI was ruled out using the modified ESC algorithm to those that had previously been reported using the same cut-off concentrations for a large, multinational, cohort of patients with suspected acute MI.^12^ Although we only included patients with a 0-hour hs-cTnT of <12 ng/L, the findings suggest that this protocol can be used safely in patients for whom hs-cTnT concentrations have been recorded during previous hospital visits.

The risk of MI in patients with a historical hs-cTnT value >12 ng/L and a subsequent 0-hour hs-cTnT of <12 ng/L was lower than in those with a historical hs-cTnT <12 ng/L and a delta hs-cTnT relative to the 0-hour hs-cTnT of >3 ng/L (2.2% and 4.6%, respectively). The findings suggest that temporal long-term increases of hs-cTn levels are associated with higher risks of adverse cardiovascular outcomes in the future,^14 15^ and that a lowering of hs-cTnT concentrations may conversely reduce these risks.^16^ However, whether repeated hs-cTn measurement over time could be used to improve the ability of clinicians to identify individuals who are at high risk of cardiovascular disease, or to monitor or modify this risk, is unknown.

Patients with myocardial injury are not infrequently discharged directly from the ED if their hs-cTnT concentrations are similar to those measured during previous visits, if all other clinical assessments suggest low cardivascular risk, and particularly if the high hs-cTnT concentrations are considered to be related to other factors, such as a high age or a low estimated glomerular filtration rate (eGFR).^17^ However, it is unknown whether this practice is safe. A persistently high but nondynamic hs-cTn level is indicative of chronic myocardial injury, which is associated with a high risk of death and cardiovascular disease.^18–20^ Future studies of the prognostic value of historical hs-cTn values and their relationship with the 0-hour hs-cTnT concentration at presentation would likely help the development of clinical guidelines regarding a safe use of such information.

The risk of MI and the NPV of patients, for whom MI had been ruled out using the historical-hs-cTnT algorithm, were slightly higher among those who had had their historical hs-cTnT concentrations measured <1 year before the index visit compared with those with older recorded hs-cTnT concentrations. This suggests that the time when the historical measurement was made may be clinically relevant in the evaluation of its usefulness. Additionally, the potential importance of the number of measurements, the time between measurements, and the changes over time should be further explored.

### Strengths

We have studied a large cohort of patients who had historical hs-cTnT concentrations recorded in their medical records. The large size of the study sample also enabled us to perform analyses of the diagnostic performance of algorithms that incorporated these values with high precision separately in men and women, and to conduct other appropriate subgroup analyses.

The healthcare registers from which the follow-up data were obtained for this study have high validity and virtually complete nationwide coverage.^9 10^ Furthermore, we believe that the generalisability of the study findings to other hospitals in Sweden and to healthcare settings in other countries with a similar healthcare system is high.

### Study limitations

We included only patients with a 0-hour hs-cTnT of <12 ng/L; therefore, the generalisability of the results is limited to patients with a low hs-cTnT concentration at ED presentation.

The study period started in 2012, when the ESC 0/1-hour algorithm had not yet been included in the clinical guidelines for the management of acute coronary syndromes in patients presenting without persistent ST-segment elevation. At that time, the use of a 0/3-hour rule-out protocol was recommended.^21^ Changes in patient management guidance during the study period may also have influenced the results. Subsequently, this may have limited the generalisability of the study findings, although one may expect that it would have mainly affected the performance of the modified ESC algorithm, and not the historical-hs-cTnT algorithm.

For patients with hs-cTnT concentrations <5 ng/L, we assigned a level of 5 ng/L to calculate delta values. Furthermore, we did not have information regarding the time between the symptom onset and the 0-hour hs-cTnT measurement. According to the ESC guidelines, patients could be triaged toward direct rule-out of MI if the 0-hour hs-cTnT concentration is <5 ng/L and the onset of symptoms is >3 hour after presentation.^4 22–24^ However, because we restricted our study population to patients who had a second hs-cTnT level measured within 3.5 hours, all the included patients who had a 0-hour hs-cTnT <5 ng/L also had a clinical indication for the repeat analysis of hs-cTnT.

Although the standardised triage module for classifying patient symptoms at presentation was the same used at all sites during the study period, some misclassification may have occurred. However, it is likely that misclassification rates were similar in patients evaluated and ruled-out according to either of the two algorithms.

This was an observational study of data obtained during routine clinical practice. Therefore, patients were not managed according to a prespecified study protocol, such as the ESC 0/1-hour algorithm. Consequently, the findings should be interpreted with caution, and the feasability, efficacy and compliance with such protocols should be further investigated. Finally, we did not have information regarding electrocardiographic findings or those of other cardiac investigations.

## Conclusions

Using a large cohort of patients with chest pain in the ED who had a hs-cTnT level of <12 ng/L at presentation, we found that the risk of MI was minimal if historical hs-cTnT concentrations were also <12 ng/L and the delta hs-cTnT between these concentrations was <3 ng/L. These findings indicate that historical hs-cTnT concentrations may be of prognostic value and its potential use should be further investigated.

## Data Availability

Data are available on reasonable request. All data relevant to the study are included in the article or uploaded as online supplemental information. The dataset was anonymised, so that no unique patient could be identified. No permitted commercial reuse of data.
