# Supplementary material for: Use of historical high-sensitivity cardiac troponin T levels to rule out myocardial infarction
Source: Open Heart. 2021 May 14;8(1):e001682. doi: 10.1136/openhrt-2021-001682 (PMC8127977; doi:10.1136/openhrt-2021-001682)
Supplement: Supplementary data [file openhrt-2021-001682supp001.pdf]

# SUPPLEMENTAL MATERIAL

## Use of Historical High-Sensitivity Cardiac Troponin T Levels to Rule Out Myocardial Infarction

Andreas Roos, MD, PhD[a,b]; Martin J Holzmann, MD, PhD[a,b]

[a]Department of Medicine, Karolinska Institute, Solna, Stockholm, Sweden

[b]Department of Emergency and Reparative Medicine, Karolinska University Hospital, Huddinge, Stockholm, Sweden.

**Address for correspondence:** Andreas Roos, Department of Emergency and Reparative Medicine, Karolinska University Hospital, Huddinge, SE-141 86 Stockholm, Sweden.

E-mail: andreas.roos@sll.se. Telephone: 0046-709944858, Fax: +46-8-58585111.

## Table of contents

Supplemental Table 1. 1

Supplemental Table 2. 2

Supplemental Table 3 3

Supplemental Table 4. 4

Supplemental Table 5. 5

Supplemental Table 6. 6

Supplemental Table 7. 7

Characteristics of the hospitals included in the study 9



Roos A, Holzmann MJ. *Open Heart* 2022; 8:e001682. doi: 10.1136/openhrt-2021-001682

## Supplemental Table 1. Details of the selection of the study population

| | Hospitals included in the study | | | | | | | | | |
|---|---|---|---|---|---|---|---|---|---|---|
| | Danderyd Hospital | KS-Solna | KS-Huddinge | St. Görans Hospital | SÖS | Södertälje Hospital | Sahlgrenska University Hospital | Östra Hospital | Mölndal Hospital | **Total number** |
| Location, city | Stockholm | Stockholm | Stockholm | Stockholm | Stockholm | Stockholm | Gothenburg | Gothenburg | Gothenburg | |
| Number of visits for chest pain in patients ≥35 years of age | 32,185 | 15,705 | 22,025 | 25,614 | 37,233 | 8,267 | 19,371 | 17,961 | 8,260 | 186,621 |
| Patients with STEMI | 355 | 151 | 224 | 255 | 471 | 90 | 167 | 167 | 87 | 1,967 |
| Number of visits for chest pain with hs-cTnT measurement | 28,821 | 14,360 | 20,191 | 14,466 | 33,446 | 5,137 | 18,108 | 15,232 | 6,432 | 156,193 |
| Patients with a 0-h hs-cTnT level and a second hs-cTnT value measured between 45 min to ≤3.5 h | 7,109 | 4,910 | 2,549 | 1,595 | 4,527 | 276 | 8,606 | 6,954 | 1,964 | 38,490 |
| Patients with a 0-h hs-cTnT <12 ng/l | 4,690 | 3,210 | 1,412 | 1,072 | 2,735 | 189 | 4,909 | 3,219 | 847 | 22,283 |
| Patients with ≥1 historical hs-cTnT level available from a previous visit for any reason | 1,739 | 1,315 | 637 | 384 | 1,046 | 67 | 1,799 | 1,166 | 279 | 8,432 |

Abbreviations: Hs-cTnT: high-sensitivity cardiac troponin t; KS-Huddinge: Karolinska University Hospital, Huddinge; KS-Solna: Karolinska University Hospital, Solna; STEMI: ST-segment elevation myocardial infarction; SÖS: Stockholm South General Hospital.



Roos A, Holzmann MJ. *Open Heart* 2022; 8:e001682. doi: 10.1136/openhrt-2021-001682

**Supplemental Table 2. Incidence of myocardial infarction**

| | | | Historical hs-cTnT concentration | | | |
|---|---|---|---|---|---|---|
| | | | >12 ng/l | <12 ng/l | | |
| | | **Delta hs-cTnT*** | any | <3 ng/l | >3 ng/l | **Total** |
| **Delta hs-TnT†** | <3 ng/l | MI ≤30 days, n | 10/1202 | 19/6503 | 6/395 | 35/8100 |
| | | 30-day MI risk (95% CI) | 0.8% (0.4–1.6) | 0.3% (0.2–0.5) | 1.5% (0.6–3.4) | 0.4% (0.3–0.6) |
| | >3 ng/l | MI ≤30 days, n | 18/92 | 17/197 | 14/43 | 49/332 |
| | | 30-day MI risk (95% CI) | 19.6% (12.3–29.4) | 8.6% (5.3–13.7) | 32.6% (19.5–48.7) | 14.8% (11.2–19.1) |
| | **Total** | MI ≤30 days, n | 28/1294 | 36/6700 | 20/438 | 84/8432 |
| | | 30-day MI risk (95% CI) | 2.2% (1.5–3.2) | 0.5% (0.4–0.8) | 4.6% (2.9–7.1) | 1.0% (0.8–1.2) |

*Change between the historical hs-cTnT and 0-h hs-cTnT level. †Change between the 0-h hs-cTnT and second hs-cTnT level. The **green** and yellow colours represent triage toward rule-out using the modified ESC algorithm and the historical-hs-cTnT algorithm, respectively. Abbreviation: hs-cTnT: high-sensitivity cardiac troponin t.

BMJ Publishing Group Limited (BMJ) disclaims all liability and responsibility arising from any reliance placed on this supplemental material which has been supplied by the author(s)


**Supplemental Table 3. Performance of a modified ESC algorithm and a historical-hs-cTnT algorithm with the use of a historical hs-cTnT value as the 0-h value to rule out myocardial infarction and for the prediction of all-cause mortality, stratified by sex**

| | **Men** | **Women** |
|---|---|---|
| Number of eligible patients | 4,269 | 4,163 |
| MI ≤30 days after the index visit, n (%) | 55 (1.3) | 29 (0.7) |
| **Algorithm using hs-cTnT measured at the same visit (modified ESC algorithm)<br>Myocardial infarction** | | |
| ***Rule-out*** | | |
| Number of patients ruled-out, n (%) | 4,109 (96) | 3,991 (96) |
| 30-day risk of MI (95 CI%) | 0.6% (0.4–0.9) | 0.3% (0.1–0.5) |
| NPV, % (95 CI%) | 99.4 (99.1–99.6) | 99.7 (99.5–99.9) |
| LR⁻ (95 CI%) | 0.47 (0.23-0.61) | 0.36 (0.09-0.56) |
| Sensitivity, % (95 CI%) | 54.5 (40.7–67.8) | 65.5 (45.7–81.4) |
| **Algorithm using a historical hs-cTnT value as the 0-h hs-cTnT value (historical-hs-cTnT algorithm)<br>Myocardial infarction** | | |
| ***Rule-out*** | | |
| Number of patients ruled-out, n (%) | 3,270 (77) | 3,430 (82) |
| 30-day risk of MI (95 CI%) | 0.9% (0.6–1.3) | 0.2% (0.1–0.5) |
| NPV, % (95 CI%) | 99.1 (98.7–99.4) | 99.8 (99.5–99.9) |
| LR⁻ (95 CI%) | 0.66 (0.35-1.00) | 0.33 (0.09-0.57) |
| Sensitivity, % (95 CI%) | 49.1 (35.5-62.8) | 72.4 (52.5-86.6) |
| **Algorithm using hs-cTnT measured at the same visit (modified ESC algorithm)<br>All-cause mortality** | | |
| ***Rule-out*** | | |
| 30-day risk of all-cause mortality (95% CI) | 0.1% (0.0–0.2) | 0.2% (0.1–0.4) |
| NPV, % (95% CI) | 99.9 (99.8–100) | 99.8 (99.6–99.9) |
| LR⁻ (95% CI) | 1.04 (0.08-1.06) | 0.94 (0.23-1.04) |
| Sensitivity, % (95 CI%) | 0 (0.0–69.0) | 10.0 (0.5–45.9) |
| **Algorithm using a historical hs-cTnT value as the 0-h hs-cTnT value (historical-hs-cTnT algorithm)<br>All-cause mortality** | | |
| ***Rule-out*** | | |
| 30-day risk of all-cause mortality (95% CI) | 0% (0.0–0.2) | 0.1% (0.0–0.3) |
| NPV, % (95% CI) | 100 (99.8–100) | 99.9 (99.7–100) |
| LR⁻ (95% CI) | 0.44 (0.01-1.22) | 0.36 (0.03-1.00) |
| Sensitivity (95% CI) | 66.7 (12.5-98.2) | 70.0 (35.4-91.9) |

Abbreviations: CI: confidence interval; hs-cTnT: high-sensitivity cardiac troponin t; LR−: negative likelihood ratio; MI: myocardial infarction; NPV: negative predictive value.



Roos A, Holzmann MJ. *Open Heart* 2022; 8:e001682. doi: 10.1136/openhrt-2021-001682

**Supplemental Table 4. All-cause mortality**

| | | | Historical hs-cTnT concentration | | | |
|---|---|---|---|---|---|---|
| | | | >12 ng/l | <12 ng/l | | |
| | | **Delta hs-cTnT*** | any | <3 ng/l | >3 ng/l | **Total** |
| **Delta hs-TnT†** | <3 ng/l | Death ≤30 days, n | 5/1202 | 4/6503 | 3/395 | 12/8100 |
| | | 30-day all-cause mortality risk (95% CI) | 0.4% (0.2–1.0) | 0.1% (0.0–0.2) | 0.8% (0.2–2.4) | 0.1% (0.1–0.3) |
| | >3 ng/l | Death ≤30 days, n | 1/92 | 0/197 | 0/43 | 1/332 |
| | | 30-day all-cause mortality risk (95% CI) | 1.1% (0.1–6.8) | 0% (0.0–2.4) | 0% (0.0–10.2) | 0.3% (0.0–1.9) |
| | **Total** | MI ≤30 days, n | 6/1294 | 4/6700 | 3/438 | 13/8432 |
| | | 30-day all-cause mortality risk (95% CI) | 0.5% (0.2–1.1) | 0.1% (0.0–0.2) | 0.7% (0.2–2.2) | 0.2% (0.1–0.3) |

*Change between the historical hs-cTnT and 0-h hs-cTnT level. †Change between the 0-h hs-cTnT and second hs-cTnT level. The **green** and **yellow** colours represent triage toward rule-out using the modified ESC algorithm and the historical-hs-cTnT algorithm, respectively. Abbreviation: hs-cTnT: high-sensitivity cardiac troponin t.



Roos A, Holzmann MJ. *Open Heart* 2022; 8:e001682. doi: 10.1136/openhrt-2021-001682

**Supplemental Table 5. Performance of a modified ESC algorithm and a historical-hs-cTnT algorithm for rule out of myocardial infarction, stratified according to period of time before the remeasurement of hs-cTnT concentration**

| **Algorithm using hs-cTnT measured at the same visit (modified ESC algorithm)**<br>**Early resampling group: second hs-cTnT concentration measured between 45 min and <2 h after the 0-h hs-cTnT concentration** | | | |
|---|---|---|---|
| | **All patients** | **Men** | **Women** |
| Number of eligible patients | 3,847 | 1,932 | 1,915 |
| MI ≤30 days after the index visit, n (%) | 26 (0.7) | 20 (1.0) | 6 (0.3) |
| ***Rule-out*** | | | |
| Number of patients ruled-out, n (%) | 3,734 (97) | 1,870 (97) | 1,864 (97) |
| 30-day risk of MI (95% CI) | 0.3% (0.1–0.5) | 0.4% (0.2–0.9) | 0.1% (0.0–0.4) |
| NPV, % (95% CI) | 99.7 (99.5–99.9) | 99.6 (99.1–99.8) | 99.9 (99.6–100.0) |
| Sensitivity, % (95% CI) | 61.5 (40.7–79.1) | 60.0 (36.4–80.0) | 66.7 (24.1–94.0) |
| **Algorithm using hs-cTnT measured at the same visit (modified ESC algorithm)**<br>**Late resampling group: second hs-cTnT concentration measured between 2 h and ≤3.5 h after the 0-h hs-cTnT concentration** | | | |
| | **All patients** | **Men** | **Women** |
| Number of eligible patients | 4,585 | 2,337 | 2,248 |
| MI ≤30 days after the index visit, n (%) | 58 (1.3) | 35 (1.5) | 23 (1.0) |
| ***Rule-out*** | | | |
| Number of patients ruled-out, n (%) | 4,366 (95) | 2,239 (96) | 2,127 (95) |
| 30-day risk of MI (95% CI) | 0.6% (0.4–0.9) | 0.8% (0.5–1.2) | 0.4% (0.2–0.8) |
| NPV, % (95% CI) | 99.4 (99.1–99.6) | 99.2 (98.8–99.5) | 99.6 (99.2–99.8) |
| Sensitivity, % (95% CI) | 56.9 (43.3–69.6) | 51.4 (34.3–68.3) | 65.2 (42.8–82.8) |

| **Algorithm using historical hs-cTnT concentration as the 0-h hs-cTnT concentration (historical-hs-cTnT algorithm)**<br>**Historical values measured at various times before the 0-h hs-cTnT concentration** | | |
|---|---|---|
| | **≤365 days before 0-h hs-cTnT** | **>365 days before 0-h hs-cTnT** |
| Number of eligible patients | 5,490 | 2,942 |
| MI ≤30 days after the index visit, n (%) | 54 (1.0) | 30 (1.0) |
| ***Rule-out*** | | |
| Number of patients ruled-out, n (%) | 4,325 (94) | 2,375 (93) |
| 30-day risk of MI, (95% CI) | 0.4% (0.3–0.7) | 0.8% (0.5–1.2) |
| NPV, % (95% CI) | 99.6 (99.3–99.7) | 99.2 (98.8–99.5) |
| Sensitivity, % (95% CI) | 66.7 (52.4-78.5) | 40.0 (23.2-59.2) |

Abbreviations: CI: confidence interval; hs-cTnT: high-sensitivity cardiac troponin t; MI: myocardial infarction; NPV: negative predictive value.



Roos A, Holzmann MJ. *Open Heart* 2022; 8:e001682. doi: 10.1136/openhrt-2021-001682

**Supplemental Table 6. Performances of a modified ESC algorithm and a historical-hs-cTnT algorithm for the prediction of all-cause mortality, stratified according to the period of time to remeasurement of the hs-cTnT concentration**

| **Algorithm using hs-cTnT measured at the same visit (modified ESC algorithm)**<br>**Early resampling group: second hs-cTnT concentration measured between 45 min and <2 h after the 0-h hs-cTnT concentration** | | | |
|---|---|---|---|
| | **All patients** | **Men** | **Women** |
| Number of eligible patients | 3,847 | 1,932 | 1,915 |
| Death ≤30 days after the index visit, n (%) | 5 (0.1) | 2 (0.1) | 3 (0.2) |
| ***Rule-out*** | | | |
| 30-day risk of all-cause mortality (95% CI) | 0.1% (0.0–0.3) | 0.1% (0.0–0.4) | 0.2% (0.0–0.5) |
| NPV, % (95% CI) | 99.9 (99.7–100.0) | 99.9 (99.6–100.0) | 99.8 (99.5–100.0) |
| Sensitivity, % (95% CI) | 0.0 (0.0–53.7) | 0.0 (0.0–80.2) | 0.0 (0.0–69.0) |
| **Algorithm using hs-cTnT measured at the same visit (modified ESC algorithm)**<br>**Late resampling group: second hs-cTnT concentration measured between 2 h and ≤3.5 h after the 0-h hs-cTnT concentration** | | | |
| | **All patients** | **Men** | **Women** |
| Number of eligible patients | 4,585 | 2,337 | 2,248 |
| Death ≤30 days after the index visit, n (%) | 8 (0.2) | 1 (0.0) | 7 (0.3) |
| ***Rule-out*** | | | |
| 30-day risk of all-cause mortality (95% CI) | 0.2% (0.1–0.3) | 0.0% (0.0–0.3) | 0.3% (0.1–0.6) |
| NPV, % (95% CI) | 99.8 (99.7–99.9) | 100.0 (99.7–100.0) | 99.7 (99.4–99.9) |
| Sensitivity, % (95% CI) | 12.5 (0.7–53.3) | 0.0 (0.0–94.5) | 14.3 (0.8–58.0) |

| **Algorithm using a historical hs-cTnT value as the 0-h hs-cTnT value (historical-hs-cTnT algorithm)**<br>**Historical values measured at various times before the 0-h hs-cTnT concentration** | | |
|---|---|---|
| | **≤365 days before the admission hs-cTnT measurement** | **>365 days before the admission hs-cTnT measurement** |
| Number of eligible patients | 5,490 | 2,942 |
| Death ≤30 days after the index visit, n (%) | 11 (0.2) | 2 (0.1) |
| ***Rule-out*** | | |
| 30-day risk of all-cause mortality (95% CI) | 0.1% (0.0–0.2) | 0.0% (0.0–0.3) |
| NPV, % (95% CI) | 99.9 (99.8–100.0) | 100.0 (99.7–100.0) |
| Sensitivity, % (95% CI) | 72.7 (39.3-92.7) | 50.0 (2.7-97.3) |

Abbreviations: CI: confidence interval; hs-cTnT: high-sensitivity cardiac troponin t; NPV: negative predictive value.



Roos A, Holzmann MJ. *Open Heart* 2022; 8:e001682. doi: 10.1136/openhrt-2021-001682

**Supplemental Table 7. Performance of a historical-hs-cTnT algorithm for rule out of myocardial infarction and prediction of all-cause mortality, including patients with only one hs-cTnT concentration measured during the visit**

**Algorithm using a historical hs-cTnT value as the 0-h hs-cTnT value (historical-hs-cTnT algorithm)**
**Myocardial infarction**

| | **All patients** | **Men** | **Women** |
|---|---|---|---|
| Number of eligible patients | 34,560 | 16,939 | 17,621 |
| MI ≤30 days after the index visit, n (%) | 252 (0.7) | 163 (1.0) | 89 (0.5) |
| ***Rule-out*** | | | |
| Number of patients ruled-out, n (%) | 28,393 (82) | 13,486 (80) | 14,907 (85) |
| 30-day risk of MI (95% CI) | 0.4 (0.4-0.5) | 0.6 (0.5-0.8) | 0.3 (0.2-0.4) |
| NPV, % (95% CI) | 99.6 (99.5-99.6) | 99.4 (99.2-99.5) | 99.7 (99.6-99.8) |
| Sensitivity, % (95% CI) | 51.2 (44.9-57.5) | 48.5 (40.6-56.4) | 56.2 (45.3-66.5) |

**Algorithm using a historical hs-cTnT value as the 0-h hs-cTnT value (historical-hs-cTnT algorithm)**
**Myocardial infarction**
**Historical values measured at various times before the 0-h hs-cTnT concentration**

| | **≤365 days before the admission hs-cTnT measurement** | **>365 days before the admission hs-cTnT measurement** |
|---|---|---|
| Number of eligible patients | 24,527 | 10,033 |
| MI ≤30 days after the index visit, n (%) | 186 (0.8) | 66 (0.7) |
| ***Rule-out*** | | |
| Number of patients ruled-out, n (%) | 20,022 (82) | 8,371 (83) |
| 30-day risk of MI (95% CI) | 0.4 (0.3-0.5) | 0.5 (0.3-0.7) |
| NPV, % (95% CI) | 99.6 (99.5-99.7) | 99.5 (99.3-99.7) |
| Sensitivity, % (95% CI) | 55.4 (47.9-62.6) | 39.4 (27.8-52.2) |

**Algorithm using a historical hs-cTnT value as the 0-h hs-cTnT value (historical-hs-cTnT algorithm)**
**All-cause mortality**

| | **All patients** | **Men** | **Women** |
|---|---|---|---|
| Number of eligible patients | 34,560 | 16,939 | 17,621 |
| Death ≤30 days after the index visit, n (%) | 64 (0.2) | 24 (0.1) | 40 (0.2) |
| ***Rule-out*** | | | |
| 30-day risk of all-cause mortality (95% CI) | 0.2% (0.1-0.2) | 0.1% (0.1-0.2) | 0.2% (0.1-0.3) |
| NPV, % (95% CI) | 99.8 (99.8-99.9) | 99.9 (99.8-99.9) | 99.8 (99.7-99.9) |
| Sensitivity, % (95% CI) | 29.7 (19.2-42.6) | 25.0 (10.6-47.1) | 32.5 (19.1-49.2) |

**Algorithm using a historical hs-cTnT value as the 0-h hs-cTnT value (historical-hs-cTnT algorithm)**
**All-cause mortality**
**Historical values measured at various times before the 0-h hs-cTnT concentration**



Roos A, Holzmann MJ. *Open Heart* 2022; 8:e001682. doi: 10.1136/openhrt-2021-001682

| | **≤365 days before the admission hs-cTnT measurement** | **>365 days before the admission hs-cTnT measurement** |
|---|---|---|
| Number of eligible patients | 24,527 | 10,033 |
| Death ≤30 days after the index visit, n (%) | 49 (0.2) | 15 (0.1) |
| ***Rule-out*** | | |
| 30-day risk of all-cause mortality (95% CI) | 0.2 (0.1-0.2) | 0.1 (0.1-0.3) |
| NPV, % (95% CI) | 99.8 (99.8-99.9) | 99.9 (99.7-99.9) |
| Sensitivity, % (95% CI) | 32.7 (20.4-47.7) | 20.0 (5.3-48.6) |

Abbreviations: CI: confidence interval; hs-cTnT: high-sensitivity cardiac troponin t; NPV: negative predictive value.



Roos A, Holzmann MJ. *Open Heart* 2022; 8:e001682. doi: 10.1136/openhrt-2021-001682

## Characteristics of the hospitals included in the study

Patients were included from the following nine hospitals in Sweden.

**Karolinska University Hospital, Solna, Stockholm, and Karolinska University Hospital Huddinge, Stockholm**

Karolinska University Hospital is located at two sites, 22 km apart, in Solna and Huddinge. Karolinska University Hospital Solna is situated in the northern part of Stockholm city, and has a yearly attendance at the ED of approximately 74,000. The other site is located south of Stockholm in Huddinge municipality, which is administered by Stockholm County Council. The annual number of visits to the ED in Huddinge is approximately 75,000. During the study period, on-site coronary angiography and percutaneous coronary intervention (PCI) were only available at Huddinge during office hours, but at all times at Solna.

**Sahlgrenska University Hospital, Sahlgrenska, Östra and Mölndal, Gothenburg**

Sahlgrenska University Hospital was founded in 1997 when three hospitals merged: Sahlgrenska Hospital, Östra Hospital and Mölndal Hospital. All the sites are located in the city of Gothenburg, and the hospital has a total capacity of 2,000 beds. In total, there are approximately 106,000 visits per year to the adult ED.

**St. Görans Hospital, Stockholm**

St Görans Hospital is centrally located in Stockholm city, and had approximately 77,000 annual visits to the ED during the study period. During this period, on-site coronary angiography and PCI were only available during office hours.

**Stockholm South General Hospital, Stockholm**

Stockholm South General Hospital is located in the southern part of Stockholm city, and has the largest emergency care unit in the Nordic region. The annual number of visits to the ED is approximately 120,000. At the time of the study, coronary angiography was only available during office hours.

**Södertälje Hospital, Stockholm**

Södertälje Hospital is located outside Stockholm city, in Södertälje municipality, but this is administered by Stockholm County Council. This hospital has the lowest yearly ED attendance (approximately 32,000) of the hospitals in this study. Södertälje Hospital is the only hospital within Stockholm County that did not provide coronary angiography on-site. Therefore, patients treated at Södertälje Hospital who were in need of a coronary angiography, with or without percutaneous coronary intervention (PCI), were transferred to one of the other hospitals in Stockholm.



Roos A, Holzmann MJ. *Open Heart* 2022; 8:e001682. doi: 10.1136/openhrt-2021-001682
